# Percutaneous Endoscopic Sigmoidopexy for Recurrent Sigmoid Volvulus: A Systematic Review of Efficacy, Safety, and Clinical Outcomes

**DOI:** 10.7759/cureus.111400

**Published:** 2026-06-23

**Authors:** Mohammad Qaher Rasully, Wahidullah Dost, Basirullah Amn, Tawhidullah Dost, Wahida Ali, Kamila Dost, Jamaluddin Niazi, Maseha Sayer, Rana Sarhadi Jamal, Farzad Qasemi

**Affiliations:** 1 General Surgery, Jamhuriat Hospital, Kabul, AFG; 2 Surgery/Medicine, Kabul University of Medical Sciences, Kabul, AFG; 3 Faculty of Medicine, Nangarhar University, Jalalabad, AFG; 4 Epidemiology and Biostatistics, Ministry of Public Health, Kabul, AFG; 5 Global Health Entrepreneurship, Institute of Science Tokyo, Tokyo, JPN; 6 Cardiovascular Surgery, Punjab Institute of Cardiology, Lahore, PAK

**Keywords:** colonic volvulus, endoscopic fixation, high-risk patients, minimally invasive procedure, percutaneous endoscopic sigmoidopexy, prevention, recurrence, sigmoid volvulus, systematic review, t-fasteners

## Abstract

Sigmoid volvulus represents a significant clinical challenge, particularly in elderly patients with multiple comorbidities who experience recurrent episodes following initial endoscopic decompression. While definitive surgical resection remains the gold standard for preventing recurrence, many patients are unsuitable for major abdominal surgery due to advanced age, medical frailty, or elevated surgical risk. Percutaneous endoscopic sigmoidopexy has emerged as a minimally invasive alternative that involves endoscopically guided fixation of the sigmoid colon to the anterior abdominal wall, thereby restricting colonic mobility and preventing the anatomical conditions necessary for volvulus formation. This systematic review comprehensively evaluates the available evidence on this technique for preventing recurrent sigmoid volvulus. Following the Preferred Reporting Items for Systematic Reviews and Meta-Analyses guidelines, we searched major databases and identified five studies comprising fourteen patients who underwent the procedure. The patient population was uniformly elderly and medically complex, with multiple prior volvulus episodes. Technical approaches varied considerably across studies, including different fixation devices, numbers of anchors, spatial configurations, and use of fluoroscopic guidance. Notably, no recurrences were documented during follow-up, though duration varied markedly across studies. Based on this limited sample, the complication profile was generally favorable, with most adverse events being minor and self-limiting, though procedure-related mortality occurred in patients with severe underlying comorbidities. While the evidence base remains limited to small case series and reports, the findings suggest that percutaneous endoscopic sigmoidopexy may represent a viable option for carefully selected high-risk patients with recurrent sigmoid volvulus who are unsuitable for or decline definitive surgical intervention.

## Introduction and background

Sigmoid volvulus represents a significant cause of large bowel obstruction worldwide, characterized by the abnormal twisting of the sigmoid colon around its mesentery. This condition accounts for approximately 60-75% of all colonic volvulus cases and represents a considerable proportion of intestinal obstructions globally, with marked geographic variation in incidence and presentation patterns [1]. In Western societies, sigmoid volvulus constitutes approximately 2-5% of all intestinal obstructions, while in endemic regions spanning the “volvulus belt” of Africa, the Middle East, and parts of South America, it may account for up to 50% of large bowel obstructions [2]. The community-based incidence in the United States is approximately 1.67 per 100,000 persons annually, with higher rates observed in institutionalized populations [3].

The clinical presentation of sigmoid volvulus typically occurs in elderly, institutionalized patients with multiple comorbidities, particularly those with chronic constipation, neuropsychiatric disorders, or prolonged use of psychotropic medications that affect colonic motility. In Western countries, the condition predominantly affects individuals in their eighth decade of life, while in endemic regions, patients tend to be younger and predominantly male [4]. Risk factors include an elongated sigmoid colon (dolichosigmoid) with a narrow mesenteric base, chronic constipation, high-fiber diets leading to colonic overloading, and conditions such as Hirschsprung’s disease or Chagas disease. The pathophysiology involves twisting of the sigmoid colon around its mesenteric axis, resulting in closed-loop obstruction that can progress to vascular compromise, bowel ischemia, and potentially perforation if left untreated.

Current management strategies for sigmoid volvulus emphasize initial endoscopic decompression in the absence of peritonitis, ischemia, or perforation. Flexible sigmoidoscopy has become the primary therapeutic approach, achieving successful decompression rates typically reported between 70% and 90% in various series [5]. However, the major limitation of endoscopic decompression alone is the substantial risk of recurrence, with studies reporting recurrence rates between 40% and 90% following successful initial decompression [6]. The median time to recurrence ranges from approximately two to three months, with some studies documenting recurrence in up to 84% of patients who undergo conservative management alone [7]. Consequently, current consensus guidelines strongly recommend definitive surgical intervention, preferably sigmoid colectomy with primary anastomosis, to be performed during the index admission or shortly thereafter to prevent recurrent episodes [5].

Despite the established benefits of surgical resection in preventing recurrence, many patients with sigmoid volvulus present significant surgical challenges. The typical patient demographic includes elderly individuals with multiple medical comorbidities, limited functional status, and elevated American Society of Anesthesiologists physical status classifications. In this population, elective sigmoid resection carries high mortality risk, while emergency surgery for complications such as ischemia or perforation is associated with substantially higher morbidity and mortality rates [8]. These sobering statistics highlight a critical therapeutic dilemma: while definitive surgery offers the best chance of preventing recurrence, a substantial proportion of patients are either deemed unsuitable for major surgery or refuse surgical intervention due to associated risks.

In response to this clinical challenge, percutaneous endoscopic sigmoidopexy (PES) has emerged as a minimally invasive alternative for preventing recurrent sigmoid volvulus in high-risk patients. This technique involves endoscopically guided fixation of the sigmoid colon to the anterior abdominal wall, thereby restricting colonic mobility and preventing the anatomical conditions necessary for volvulus formation [9]. Various technical approaches have been described, including the use of T-fasteners, anchor systems, and buried suture techniques. The procedure is typically performed under conscious sedation with local anesthesia, eliminating the need for general anesthesia and avoiding the physiological stress of major abdominal surgery. Early case reports and small series have suggested that PES may be effective in preventing recurrence with acceptable safety profiles in carefully selected patients.

Despite the apparent promise of PES as an alternative to surgical resection in high-risk patients, the current evidence base remains fragmented, consisting primarily of case reports and small case series with considerable heterogeneity in patient selection, technical approach, fixation method, and outcome reporting. Critical questions remain unanswered regarding optimal technique, durability of volvulus prevention, complication rates, and comparative effectiveness versus other management strategies. Furthermore, the role of this technique within the broader therapeutic algorithm for sigmoid volvulus management has not been clearly defined.

This systematic review aims to comprehensively evaluate the available evidence on PES for the prevention of recurrent sigmoid volvulus. By systematically identifying, appraising, and synthesizing all available studies, we seek to determine the efficacy, safety, and clinical outcomes associated with this technique. Specifically, we aim to assess recurrence rates following the procedure, characterize the spectrum and frequency of complications, evaluate technical success rates and procedural variations, and identify optimal patient selection criteria. Through this comprehensive analysis, we hope to inform clinicians’ understanding of the current evidence and its limitations regarding the potential role of PES in the management of patients with recurrent sigmoid volvulus, particularly those who are unsuitable for or decline definitive surgical intervention.

## Review

Methodology

This systematic review was conducted and reported in accordance with the Preferred Reporting Items for Systematic Reviews and Meta-Analyses (PRISMA) 2020 guidelines to ensure a transparent and structured evaluation of the available evidence on PES for recurrent sigmoid volvulus [10].

Search Strategy

A comprehensive literature search was performed across major electronic databases, including MEDLINE (via PubMed), Embase, the Cochrane Central Register of Controlled Trials (CENTRAL), and Web of Science, from January 2015 to January 2026. The search strategy combined controlled vocabulary and free-text terms related to the condition and intervention, including “sigmoid volvulus,” “recurrent sigmoid volvulus,” “percutaneous endoscopic sigmoidopexy,” “endoscopic sigmoidopexy,” and related procedural terminology. Reference lists of all eligible studies and relevant reviews were also screened manually to identify additional studies not captured through database searching.

Eligibility Criteria

Studies were considered eligible if they included adult patients diagnosed with recurrent sigmoid volvulus and evaluated PES or closely related endoscopic fixation techniques used for its prevention or management. Eligible studies were required to report clinically relevant outcomes such as procedural success, recurrence, complications, mortality, or other related outcomes. A broad range of study designs was considered, including randomized controlled trials, non-randomized studies, cohort studies, case-control studies, case series, and case reports describing the clinical application of the procedure. Studies were excluded if they were conducted in animal or in vitro settings, or if they were narrative reviews, systematic reviews, editorials, or conference abstracts lacking sufficient clinical data. Studies that did not report outcomes specific to PES were also excluded.

Study Selection

Two independent reviewers (B.A. and W.D.) screened all retrieved records. Titles and abstracts were assessed first to identify potentially relevant studies. Full-text articles of eligible records were then reviewed in detail against the predefined inclusion and exclusion criteria. Disagreements between reviewers were resolved through discussion, and consensus was reached before final inclusion.

Data Extraction

Data were extracted independently by two reviewers (K.D. and T.D.) using a predesigned data extraction form. The extracted information included study characteristics such as author, year of publication, country, and study design; patient characteristics including sample size and available demographic details; clinical information related to recurrent sigmoid volvulus; procedural details of PES; and reported outcomes, including recurrence, complications, need for additional interventions, and mortality. Any discrepancies encountered during the data extraction process were resolved through discussion between the reviewers to reach consensus.

Quality Assessment

Where applicable, methodological quality and risk of bias of included studies were assessed using appropriate tools based on study design. The methodological quality of included case reports and case series was assessed using the Joanna Briggs Institute (JBI) Critical Appraisal Checklist for Case Reports and the JBI Critical Appraisal Checklist for Case Series [11].

Data Synthesis and Analysis

Given the expected heterogeneity in study designs, patient populations, and outcome reporting, a quantitative meta-analysis was not prespecified. Findings were synthesized narratively, with results summarized in descriptive tables outlining study characteristics, procedural details, and outcomes. The evidence was interpreted in the context of current clinical practice and existing management strategies for recurrent sigmoid volvulus.

Results

Study Selection Process

**Figure 1:**
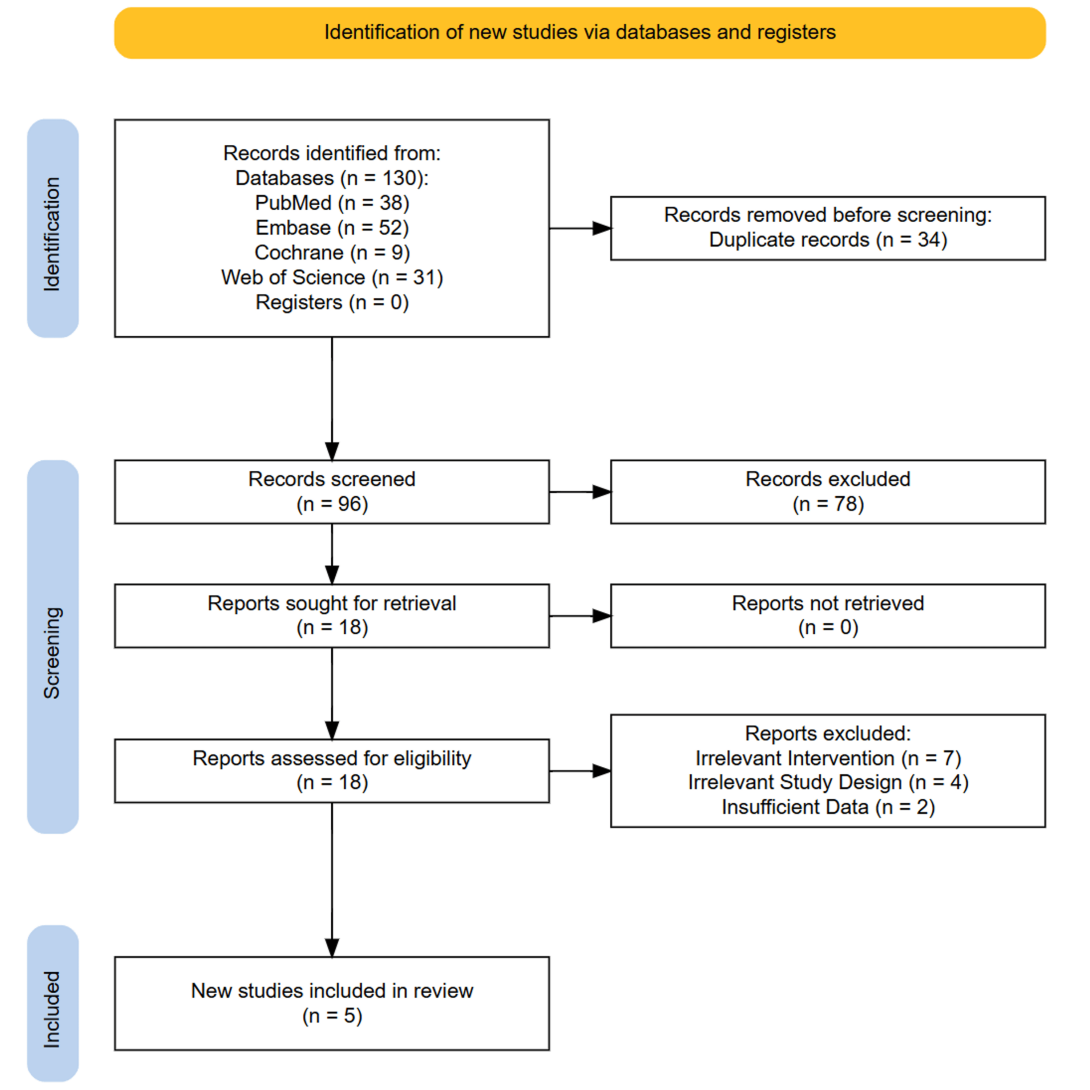
Preferred Reporting Items for Systematic Reviews and Meta-Analyses (PRISMA) diagram illustrating the study selection process.

The systematic literature search yielded a total of 130 records across four electronic databases: PubMed/MEDLINE (38 records), Embase (52 records), Cochrane CENTRAL (9 records), and Web of Science (31 records). After removing 34 duplicate records, 96 unique records remained for screening. During the initial screening phase, 78 records were excluded based on title and abstract review as they did not meet the eligibility criteria. The remaining 18 full-text articles were assessed in detail for eligibility. Following full-text review, 13 articles were excluded for the following reasons: seven studies involved non-relevant interventions, four were reviews or editorials, and two were conference abstracts with insufficient data. Ultimately, five studies met the inclusion criteria and were included in the qualitative synthesis. The included studies comprised two case series and three case reports (Figure 1).

Study Characteristics

The five included studies were published between 2015 and 2024. The studies included 14 patients who underwent PES for recurrent sigmoid volvulus. The patient population was predominantly elderly, with ages ranging from 43 to 93 years. Among the patients with reported gender, the majority were male. All studies employed endoscopic techniques for sigmoid fixation to the anterior abdominal wall, though the specific devices and technical approaches varied across studies. The fixation devices included T-fasteners from various manufacturers (Kimberly-Clark MIC G Introducer gastrostomy kit, Entuit Secure gastrointestinal suture anchor set from Cook Medical), a 2-shot anchor device from Olympus, and the Ideal PEG kit from Olympus Medical Systems with buried sutures. The number of fixation points ranged from four to eleven, with varying deployment patterns including triangular configurations. Follow-up duration varied considerably across the studies, ranging from three days to 38 months, with a median follow-up of approximately six months in the larger case series (Table 1).

**Table 1:** Characteristics and outcomes of included studies on percutaneous endoscopic sigmoidopexy for recurrent sigmoid volvulus. Y: years; M: male; F: female; BMI: body mass index; PES: percutaneous endoscopic sigmoidopexy; PEG: percutaneous endoscopic gastrostomy; mo: months; CHF: congestive heart failure; CIPO: chronic intestinal pseudo-obstruction

Author	Year	Study	Number of patients	Age/Gender	Method	Type of device used	Recurrence of volvulus?	Follow-up duration	Complications
Ito et al. [9]	2015	Case report	1	86 Y/F	PES using abdominal wall fixation with buried sutures	Ideal PEG kit (Olympus Medical Systems) - six fixation points using 2-0 non-absorbable monofilament sutures	No	30 days (the patient died from acute myocardial ischemia without recurrent volvulus)	None reported
Tin et al. [12]	2017	Case series	3	88 Y/M, 68 Y/M, 81 Y/M	PES using T-fasteners	T-fasteners from Kimberly-Clark MIC G Introducer gastrostomy kit (four fasteners deployed)	No	6 mo/9 mo/3 days	None/None/Post-procedure pneumoperitoneum (managed conservatively); died from CHF exacerbation
Imakita et al. [13]	2019	Case series	8	Median age: 72.5 Y (range: 43-93); 5 M/3 F; Median BMI: 17.9 (range: 15.6-20.2)	PES using 2-shot anchor device	2-shot anchor device (Olympus, Tokyo, Japan); average 8.8 fixation sites (range: 5-11)	No	Median: 25.5 mo (range: 4-38 mo)	One case of postoperative subcutaneous emphysema (managed conservatively)
Manasra et al. [14]	2024	Case report	1	82 Y/F	PES using T-fasteners	Entuit secure gastrointestinal suture anchor set from COOK Medical (three sets of 2-shot anchors = six anchors total, deployed in triangular/Mercedes-Benz pattern)	No	3 mo	One puncture site secured with endoscopic clip due to minor bleeding and gas bubbling. Post-procedure chronic intestinal pseudo-obstruction with persistent abdominal distension and diffuse colon dilation for ~4 weeks (treated with laxatives, enemas, and one sigmoidoscopy decompression at 2 weeks). The patient had six previous episodes of sigmoid volvulus before the procedure
Garrido et al. [15]	2023	Case report	1	85 Y/M	PES using T-fasteners	Entuit Secure systems from Cook Medical® (three fasteners in distal sigmoid + additional fixation in proximal sigmoid)	No	4 mo	Mild pneumoperitoneum (resolved), mild abdominal pain

Quality Assessment

The quality assessment revealed high adherence to reporting standards and low risk of bias within the constraints of the study design across the included studies (Table 2). Common strengths across all studies included clear intervention descriptions, appropriate outcome reporting, and adequate follow-up within the reported timeframes. Weaknesses included limited sample sizes inherent to case reports/series, lack of comparison groups, and variable follow-up duration. No study was excluded based on quality assessment given the nascent evidence base for this intervention.

**Table 2:** Quality assessment of included studies using the Joanna Briggs Institute (JBI) critical appraisal checklist.

Study	Study design	JBI score	Quality rating	Key strengths	Key limitations
Ito et al. [9]	Case report	7/8 (88%)	High	Clear demographic data; detailed intervention description; comprehensive clinical assessment; good follow-up reporting	Limited reporting of co-interventions
Tin et al. [12]	Case series	7/10 (70%)	High	Multiple patients; clear intervention protocol; adequate follow-up; adverse events reported	Unclear patient recruitment process; limited baseline characteristic details; small sample
Imakita et al. [13]	Case series	9/10 (90%)	High	Consecutive patient inclusion; standardized technique; comprehensive demographic data; clear inclusion/exclusion criteria; detailed adverse event reporting; appropriate statistical presentation	Small sample size; single center
Garrido et al. [15]	Case report	6/8 (75%)	High	Clear patient demographics; detailed procedural description; good imaging documentation; appropriate follow-up	Brief follow-up duration; limited detail on post-procedure management
Manasra et al. [14]	Case report	7/8 (88%)	High	Comprehensive patient history; detailed technical modifications; good complication reporting; discussion of persistent symptoms	Single patient; relatively short follow-up

Discussion

This systematic review identified five studies comprising 14 patients who underwent PES for recurrent sigmoid volvulus. The collective evidence demonstrates that PES is a technically feasible minimally invasive intervention for carefully selected high-risk patients, with no recurrences documented during follow-up periods ranging from 3 days to 38 months.

The patient population was uniformly elderly and medically complex. Imakita et al. (2019) reported the largest series with eight patients having a median age of 72.5 years (range = 43-93), while single-patient reports by Ito et al. (2015), Garrido et al. (2023), and Manasra et al. (2024) involved patients aged 86, 85, and 82 years, respectively [9,13-15]. All patients had substantial comorbidity burden, with Imakita et al. reporting ASA Physical Status (ASA-PS) classifications of 3-4 and a median Barthel index of 10, indicating severe functional impairment [13]. Neuropsychiatric disorders were particularly prevalent, including Alzheimer’s dementia, schizophrenia, Parkinson’s disease, and cerebrovascular accidents, conditions associated with chronic constipation and psychotropic medication use that predispose to sigmoid volvulus.

Patients had experienced multiple prior volvulus episodes (range = 2-15) over several years. Manasra et al. described a patient with six previous episodes, while the Imakita series documented 2-15 prior episodes (median = 1.5) over observation periods of 3-15 years (median = 7.5 years) [13,14]. This recurrence pattern underscores the critical therapeutic challenge: while endoscopic detorsion provides effective acute management, definitive intervention is essential to break the cycle of repeated episodes and cumulative complication risk.

Substantial heterogeneity characterized procedural techniques across studies. Three device types were employed: T-fasteners from the Kimberly-Clark MIC G Introducer kit (Tin et al.), the Entuit Secure anchor set from Cook Medical (Garrido et al.; Manasra et al.), and 2-shot anchor devices from Olympus (Imakita et al.; Ito et al.) [9,12-15]. The Olympus system, which allows subcutaneous burial of sutures, eliminates external hardware that could be inadvertently dislodged, particularly advantageous in cognitively impaired patients.

The number of fixation points varied dramatically from 4 (Tin et al.) to an average of 8.8 (range = 5-11) in the Imakita series [12,13]. Spatial configuration also differed, with Manasra et al. and Garrido et al. employing a triangular “Mercedes-Benz sign” pattern, while Tin et al. used linear two-point fixation [12,14,15]. The triangular approach has surgical logic: distributing tension across three points may reduce stress on individual anchors and minimize internal herniation risk between fixation sites. However, the optimal number and configuration remain undefined.

Fluoroscopic guidance was variably employed. Imakita et al. used radiographic observation throughout, while Manasra et al. successfully performed PES without fluoroscopy, relying on endoscopic transillumination and digital palpation [13,14]. The marked colonic dilation and low body mass index (17.9 kg/m²) facilitated transillumination. This suggests fluoroscopy may not be absolutely required in selected cases, though it likely remains essential for obese patients or those with less pronounced dilation.

All studies employed multiple confirmatory techniques to avoid visceral injury. Imakita et al. described four sequential steps: radiographic colonography, transillumination, digital palpation with endoscopic confirmation of sigmoid indentation, and exploratory puncture with a 23-gauge needle before deploying fixation devices [13]. This methodical approach reflects appropriate caution given the catastrophic consequences of inadvertently fixing small bowel or other structures.

No recurrences occurred in any of the 14 patients during reported follow-up. Imakita et al. documented no recurrences during a median follow-up of 25.5 months (range = 4-38), representing 194 patient-months [13]. This absence of reported recurrence across all 14 patients is notable given their prior treatment failure on conservative management, though it should not be interpreted as proven long-term efficacy given the small sample, variable and often short follow-up, and likely publication bias toward successful cases.

The mechanism of recurrence prevention likely involves both mechanical restriction of colonic mobility by fixation devices and formation of peritoneal adhesions. The observation by Pinedo and Kirberg (2001), cited in multiple studies, that T-fasteners could be removed at 28 days without subsequent recurrence suggests adhesions play a significant role [16]. However, stable T-bar positions on CT imaging in the Imakita series over 38 months indicates mechanical fixation can also provide durable prevention [13]. Both mechanisms likely act synergistically: mechanical fixation provides immediate restraint while adhesions gradually form to maintain long-term colonic position.

The complication profile varied considerably. Imakita et al. reported only one complication among eight patients, subcutaneous emphysema that resolved conservatively [13]. No peritonitis, visceral injuries, abscesses, or wound infections occurred. These findings are consistent with an acceptable short-term safety profile in this small, selected cohort, though the sample size is insufficient to reliably estimate rare or late complication rates. Pneumoperitoneum was documented in multiple studies. Garrido et al. noted mild pneumoperitoneum requiring only observation, while Tin et al. described extensive pneumoperitoneum requiring percutaneous drainage [12,15]. Manasra et al. proactively secured one puncture site with an endoscopic clip after observing minor bleeding and gas bubbling, a pragmatic modification that may reduce clinically significant pneumoperitoneum [14].

Three of 14 patients died during follow-up, though deaths were attributed to underlying comorbidities rather than procedural complications. In the case by Ito et al., the patient died at 30 days from myocardial ischemia, and one patient in the Tin et al. series died at three days from congestive heart failure exacerbation, both without volvulus recurrence [9,12]. These deaths underscore the medical frailty of this population and highlight that while PES prevents volvulus recurrence, it does not mitigate substantial mortality risk from comorbidities. Importantly, Manasra et al. described post-procedure chronic intestinal pseudo-obstruction lasting approximately four weeks, requiring laxatives, enemas, and sigmoidoscopic decompression [14]. This observation tempers expectations: while PES prevents mechanical volvulus recurrence, it does not address underlying colonic pathology including redundancy, dysmotility, or damage from prior ischemic episodes. Patients require counseling that ongoing constipation management may be necessary despite technically successful PES.

Clinical implications and future directions

Based on available evidence, PES appears to occupy a specific niche for carefully selected patients meeting the following criteria: recurrent sigmoid volvulus (typically ≥3 episodes) with successful prior endoscopic detorsion; high surgical risk (ASA-PS ≥3 or Barthel index <30) or explicit surgical refusal; absence of necrosis, ischemia, perforation, or peritonitis; successful detorsion and bowel preparation before PES; and anatomic feasibility with sigmoid in proximity to abdominal wall without interposed viscera. However, the evidence base suffers from critical limitations. All studies were case reports or small series totaling only 14 patients, with substantial technical heterogeneity and relatively short follow-up. Publication bias may selectively report successful cases while failures go unreported. The optimal number of fixation points, device selection, role of fluoroscopy, and timing of hardware removal remain undefined.

Future research priorities include prospective comparative studies versus PEC, repeated detorsions, or delayed surgery; technical optimization studies; long-term outcome registries; investigation in diverse populations including endemic regions and obese patients; cost-effectiveness analyses; and development of clinical practice guidelines. Until higher-quality evidence emerges, PES should be considered an experimental option offered after thorough informed consent, ideally within research protocols contributing to the evidence base.

## Conclusions

PES appears to be a technically feasible minimally invasive intervention with potential clinical utility for preventing recurrent sigmoid volvulus in carefully selected high-risk patients. The available evidence, limited to 14 patients across five small, low-level studies with heterogeneous and often short follow-up, shows no reported recurrences and no major procedural complications; however, these findings should not be interpreted as established efficacy or a confirmed safety profile. This technique may fill an important therapeutic gap for elderly, medically complex patients who experience repeated volvulus episodes but are unsuitable for definitive surgical resection due to prohibitive operative risk or patient preference. However, substantial questions remain regarding optimal technique, device selection, patient selection criteria, and long-term durability. The current evidence base is insufficient to support widespread adoption, and the procedure should be considered investigational pending higher-quality comparative studies. Clinicians should offer this intervention only after thorough informed consent discussions, ideally within research protocols that can contribute to establishing evidence-based practice guidelines for this emerging technique.
